# Investigation of the Penetration Performance of the Radial Forging Process for Wrought Aluminium Alloy

**DOI:** 10.3390/ma17092065

**Published:** 2024-04-27

**Authors:** Yongfei Wang, Linhua Xiong, Dongxiao Feng, Shengdun Zhao, Yi Guo

**Affiliations:** 1 School of Mechanical Engineering, Xi’an Jiaotong University, Xi’an 710049, China; 2 National Key Laboratory of Metal Forming Technology and Heavy Equipment, Xi’an 710049, China; 3 Xi’an Key Laboratory of Intelligent Equipment and Control, Xi’an 710049, China; 4 School of Energy and Power Engineering, Xi’an Jiaotong University, Xi’an 710049, China

**Keywords:** forging penetration, radial forging process, wrought aluminium alloy, numerical simulation, microstructure

## Abstract

With the wide application potential of wrought aluminium alloy in aerospace, automobile and electronic products, high-quality aluminium bars prepared by the radial forging (RF) process have received extensive attention. Penetration performance refers to the depth of radial plastic deformation of forgings, which is the key factor in determining the quality of forging. In this work, the penetration performance of the radial forging process for 6063 wrought aluminium bars is investigated by simulation using FORGE software. The minimum reduction amount of the hammer is calculated based on the forging penetration theory of forging. The influence of process parameters including forging ratio (FR) and billet temperature on the effective stress and hammer load in the RF process are investigated. The RF-deformed billet is then produced with the optimal process parameters obtained from the simulation results. The average grain size of aluminium alloy semi-solid spherical material is used to evaluate the forging penetration. Simulation results showed that the effective strain at the edge and the centre of the RF-deformed billet gradually increases, but the increasing speed of the effective strain at the edge becomes low. The hammer load first decreases quickly and then gradually maintains stability by increasing the FR. It is found that low billet temperature and high FR should be selected as appropriate process parameters under the allowable tonnage range of RF equipment. Under an isothermal temperature of 630 °C and a sustaining time of 10 min, the difference in the average grain dimension between the edge and the centre positions of the starting extruded blank is 186.43 μm, while the difference in the average grain dimension between the edge and the centre positions of the RF-deformed blank is 15.09 μm. The improvement ratio of penetration performance for the RF-deformed blank is obtained as 91.19%.

## 1. Introduction

Wrought aluminium alloys are characterised by low density, low cost, high strength, great plasticity, large specific strength, outstanding corrosion resistance, excellent cutting performance, superior weldability, etc. They are widely applied in aerospace, automobile and electronic products, which shows a great application potential [1,2,3]. As one type of wrought aluminium alloy, the 6xxx series wrought aluminium alloys, such as the 6063 alloy, are an Al-Mg-Si based aluminium alloy which mainly contains the α-Al matrix and the β-Mg_2_Si phase. This alloy is widely used in the automotive product, motorcycle and 3C fields due to their low mass, corrosion resistance, weldability, damping capacity and high mechanical properties [4,5]. As a common metal material in the market, wrought aluminium bars are usually prepared by the extrusion plastic forming process [6,7], while wrought aluminium alloy plates are generally produced by the cold rolling process. During the extrusion plastic forming process or the cold rolling forming process, the microstructural texture is enhanced, which can be attributed to the formation of fibrous grains resulting from the extrusion and rolling deformation [8]. Moreover, the deformation energy can be stored in the deformed alloy in the form of dislocations [9]. After the deformation from extrusion and cold rolling processes, a large number of lath-like grain structures may be formed in the wrought aluminium alloy. However, the lath-like grain structure in the microstructure of the extruded and rolled material can cause stress corrosion cracking under an alternating load, resulting in potential safety hazards [10,11,12].

Radial forging (RF) is a forming process that rapidly and synchronously forges along the radial direction of the blank with multiple (generally four) hammers to generate the plastic deformation of an intermittently fed bar or tube blank [13,14]. It has the advantages of high efficiency, excellent accuracy, great material utilisation rate and outstanding material performance improvement [15]. However, if the internal deformation is insufficient during the RF process, it might generate void and crack defects inside the forgings. Therefore, the penetration performance is an important factor for the RF process of the metal blanks.

In recent decades, the penetration performance of RF deformed blanks has received extensive attention from researchers. Sanjari et al. [16] employed finite element analysis, an artificial neural network and the Taguchi method to optimise the strain inhomogeneity in the RF process of 6082 aluminium alloy rods. Zou et al. [17] investigated the mechanical properties of ZK60 magnesium alloy processed by the RF process. They found that excellent mechanical properties could be attained after the RF process with three passes. Du et al. [18] investigated the influence of forging parameters on the penetration of the RF-deformed 25CrMo_4_ alloy steel through simulations. Results showed that the penetration performance was improved by increasing the forging ratio (FR). Yang et al. [19] studied the penetration performance based on the wrinkle evolution results of the inner surface of the 30SiMn_2_MoVA steel gun barrel produced by RF through both simulations and experiments. It was revealed that the ratio of height to width for the wrinkle linearly increased with the rise in the strain ratio. 

In summary, the investigation of the penetration performance of RF-deformed blanks was mainly carried out in three areas including the plastic strain, the stress state and microstructure research. Though the RF process has been widely used for the deformation of metal bars, little research has been performed that focuses on the penetration performance of the aluminium bar. The warm RF process with an alloy temperature of 300 °C was successfully used to deform 6063 aluminium alloy in our previous research work. However, the penetration performance of the RF-deformed alloy was not revealed [20]. 

In this work, the average grain size of semi-solid material prepared by the RF process and the isothermal heat treatment process is proposed to examine the forging penetration efficiency of an RF-deformed 6063 wrought aluminium alloy blank. The simulations of the RF process are carried out by using FORGE software with the version number of 1.1, where the minimum radial reduction value of the hammer die is calculated based on the isosceles right triangle (IRT) method. The influence of FR and blank temperature on the effective stress and hammer load in the RF process is investigated. Furthermore, the RF-deformed blank is prepared with the suggested process parameters obtained from the simulation results. The forging penetration efficiency of the RF-deformed blank is evaluated and verified based on the average grain dimension.

## 2. Materials and Methods

The commercial extruded bar with the material of 6063 wrought aluminium alloy in a T6 heat-treated state is adopted as the raw material in this study, which is purchased from the Xi’an Litong Electromechanical Equipment Manufacturing Co., Ltd., Xi’an, China. The raw material has a diameter of 100 mm and a length of 1000 mm. The specifications of the chemical elements are summarised in Table 1, which were detected by the Bruker S8 Tiger X-ray fluorescence spectrometer with the detection ranges from sub-ppm to 100%. The solidus temperature of the material is 615 °C while the liquidus temperature is 655 °C [21].

**Figure 1:**
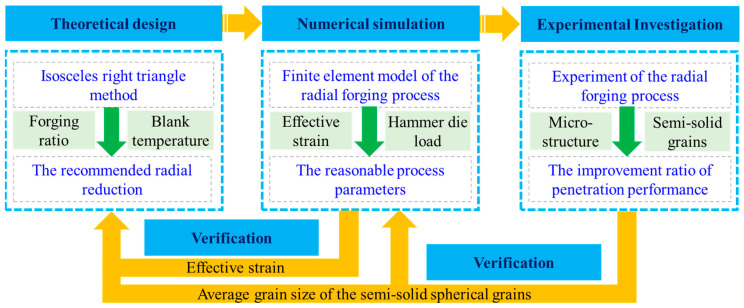
Schematic illustration for investigation of the penetration performance of the radial forging process for wrought aluminium alloy.

The research process for investigating the penetration performance of the RF process for the wrought aluminium alloy is shown in Figure 1, which can be divided into three steps. The first step is the theoretical design. The IRT method [22] is used to design the penetration performance of the wrought aluminium alloy blank. After that, the recommended radial reduction is obtained to support determining operating parameters in the RF process. The second step is the numerical simulation. The effect of FR and blank temperature on the effective stress and hammer load in the RF process is investigated to reveal appropriate process parameters. The third step is experimental investigation. The RF-deformed blank is prepared with the appropriate process parameters. The penetration performance of the RF-deformed blank is evaluated by the difference in the average grain dimension, which can further verify the rationality and correctness of the theoretical design and numerical simulation.

### 2.1. Theoretical Design for the Penetration Performance of the RF Process

**Figure 2:**
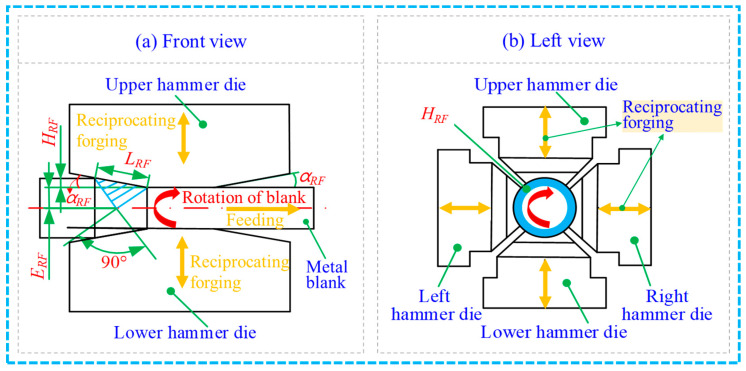
The RF process with the IRT method.

The RF process with the IRT method is illustrated in Figure 2. During the RF process, four hammer dies including upper, lower, left and right dies are radially located outside the metal blank. Numerous short strokes with fast pressings are provided to the metal blank. Simultaneously, the metal blank axially and circumferentially moves along the hammer dies. After that, the RF-deformed blank can be prepared, and its FR can be calculated using the following equation [19,22].

(1) $$FR=\frac{A_{0}\;s-{\;A}_{1}}{A_{0}}\times 100\%$$

where FR is the forging ratio, $A_{0}$ is the cross-sectional area of the initial metal blank and $A_{1}$ is the cross-sectional area of the deformed metal blanks.

The IRT method is used to design the unilateral reduction in the die. The forging penetration depth can be expressed by the following formula [23,24]:

(2) $$E_{RF}=\frac{\sqrt{2}}{2}\times L_{RF}sin\left(\frac{\pi }{4}-\alpha_{RF}\right)$$

(3) $$H_{RF}=\frac{D-d}{2}$$

(4) $$L_{RF}=\frac{H_{RF}}{\sin\alpha_{RF}}$$

where $E_{RF}$ is the penetration depth, $L_{RF}$ refers to the contact length between the hammer die and the RF-deformed blank, $H_{RF}$ refers to the unilateral reduction in the die in the radial direction, $\alpha_{RF}$ is the inclination angle of the die, *D* is the starting diameter of the blank and *d* is the diameter of the RF-deformed blank.

When the FR of the starting material is 64% (with 60 mm in the diameter of the RF-deformed blank), the penetration depth during the radial forging deformation process is larger than 30 mm. The inclination angle of the hammer dies is 4° in this work. Therefore, according to Equations (2) and (3), Equation (4) can be obtained.

(5) $$H_{RF}\geq \frac{\sqrt{2}\times E_{RF}\times \sin\alpha_{RF}}{\sin\left(45{}^{\circ}-\alpha_{RF}\right)}=4.51\;\mathrm{mm}$$

In order to ensure the uniformity of deformation between the edge and the centre of the RF-deformed 6063 aluminium alloy blank, the minimum radial reduction required for the last pass of RF deformation can be obtained as 9.02 mm based on Equation (5). Therefore, the radial reduction of 10 mm is recommended in the subsequent numerical simulation process.

### 2.2. Simulation Setting

**Figure 3:**
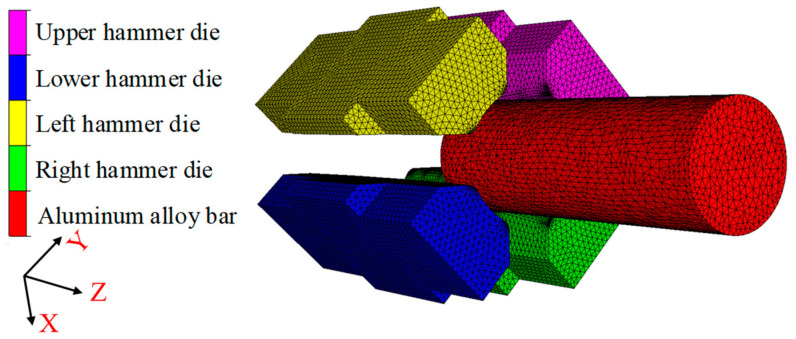
Simulation model of the RF process for the 6063 wrought aluminium alloy.

**Figure 4:**
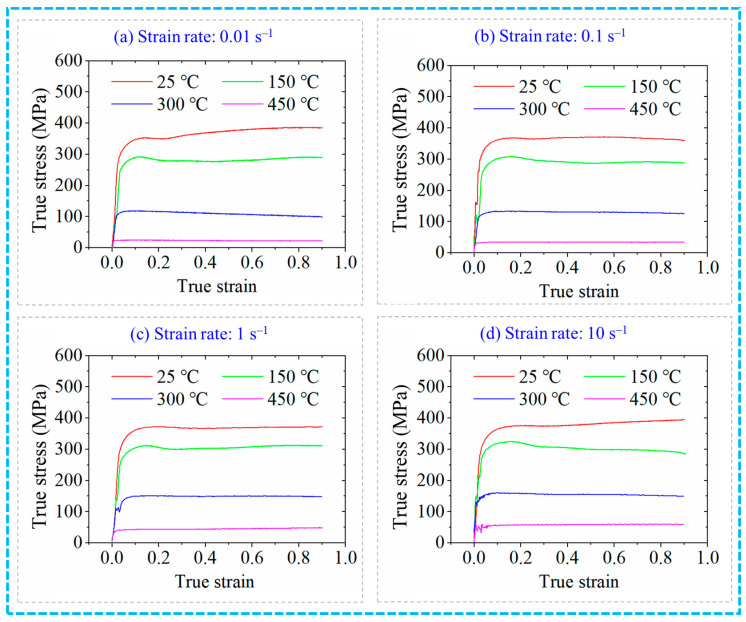
True stress–strain curves of the 6063 aluminium alloy.

The simulation model of the RF process for the 6063 wrought aluminium alloy is provided in Figure 3. The main dimension of the hammer die is 80 mm × 120 mm × 280 mm with an inclination of the hammer die of 4°. In the simulation process of RF, the material model of 6063 wrought aluminium alloy in the material library of the FORGE software is adopted, where the true stress–strain curves shown in Figure 4 reveals the relationship between the true stress and true strain under different strain rates and temperatures. The heat conductivity and specific heat capacity of this alloy are 180.2 W/m∙K and 900 J/kg∙K, respectively. The meshing of the aluminium alloy blank geometric model is carried out in FORGE software with the tetrahedral mesh. There are 111,362 total mesh elements in this work. The volume compensation is applied in the simulation for the billet. Both the ambient temperature and mould temperature are set at 25 °C. The heat transfer coefficient between the blank and the hammer die is set as 2 × 10^4^ W/(m^2^∙°C). The heat transfer coefficient between the environment and the blank or the hammer die is set as 50 W/(m^2^∙°C). The mixed friction model is used in simulations with the shear friction coefficient of 0.4 and coulomb friction coefficient of 0.2. In order to ensure the convergence of the numerical calculation, the function of repartition is enabled, the repartition step is set to 10 operation steps and the trigger condition of repartition is set to the deformation of 0.2. In the deformation process of RF, the aluminium alloy blank is axially fed while rotating. Meanwhile, it is subjected to the high-frequency-pulse multi-directional synchronous radial reciprocating forging of four hammer dies, resulting in complex geometric boundary conditions of the blank in the deformation zone. This causes periodical changes in the forming load, leading to material nonlinearity and geometric nonlinearity in blank deformation. Therefore, during the numerical simulation process, the main influence factors are considered with simplification and assumptions as shown below [25,26].

(1) The hammer die is regarded as a rigid component in the process of RF deformation, as it has a significantly smaller deformation.
(2) The elastic deformation in the metal blank is ignored in the process of RF deformation, as its value is significantly low compared to plastic deformation, which has a limited effect on the deformation.
(3) Defects and density change in the material are ignored.
(4) The weight of the material and the hammer dies are negligible.
(5) The initial material has an isotropic and uniform structure.

To further ensure the penetration performance of the RF process, multiple-pass forging is considered and carried out in the numerical simulation process, where the maximum FR of 75% (with 50 mm in the diameter of the RF-deformed blank). Therefore, the RF process is conducted with five passes: (1) from a diameter of 100 mm to 90 mm, (2) from a diameter of 90 mm to 80 mm, (3) from a diameter of 80 mm to 70 mm, (4) from a diameter of 70 mm to 60 mm; and (5) from a diameter of 60 mm to 50 mm. To our knowledge, the billet temperature has an important effect on the forming load, which is a key factor in determining whether the RF process can be realised [20]. Therefore, different blank temperatures at the start are considered in the numerical simulation process: 25 °C (ambient temperature), 150 °C, 300 °C and 450 °C. The process parameters in the numerical simulation are provided in Table 2.

### 2.3. Experimental Setup and Microstructure Analysis

**Figure 5:**
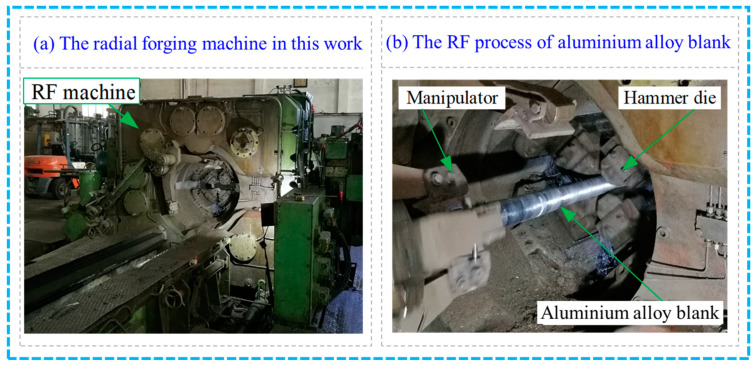
Experimental equipment photo and RF process for the RF machine.

Based on the results of the RF simulations, the RF test with different FRs was carried out for the 6063 aluminium alloy with appropriate process parameters so as to investigate and verify the forging penetration efficiency of the metal blank. Figure 5 shows the GFM SXP-16 RF machine used in this work, which includes four hammer dies and two manipulators. The forging blank diameter ranged from 30 mm to 160 mm. The maximum radial reduction and forging force were 12 mm and 200 t, respectively.

In order to investigate the penetration performance of the radial forging process for the 6063 wrought aluminium alloy, the metal specimens were cut from the edge and centre positions of both the starting material and the RF-deformed blank. The metal specimens were treated with the semi-solid isothermal heat treatment (SSIHT) process at 630 °C for 10 min to prepare the semi-solid blank. The penetration performance of the RF-deformed blank was evaluated and verified based on the analysis of the macro-morphology and metallographic structure of the RF-deformed blank as well as the difference in the average grain size of the semi-solid blank. The metal specimens were ground, polished and etched in a hydrofluoric acid solution with a concentration of 5 vol.% for a duration in the range of 90–120 s. An optical microscope (NIKON ECLIPSE LV 150N, Nikon Corporation, Tokyo, Japan) was used in the study to obtain the microstructure information. As one key index for the microstructure, the average grain size (AGS) was expressed by the following equation [27].

(6) $$AGS=\frac{\sum_{N=1}^{N}\sqrt{\frac{4A}{\pi }}}{N}$$

where A is the area of the grain and N is the grain number.

It is known that the average grain size is influenced by the FR in the RF process [20], where the FR would further affect the penetration performance. Therefore, in this study, the grain microstructure analysis is selected to indicate the penetration performance. Moreover, the SSIHT is adopted to quantitatively see the effect of the RF process on the microstructure. In terms of the influence of static recovery during the reheating, it can be argued that the recovery is the same for all different FRs as all samples are reheated under the same temperature and time conditions. Therefore, the difference in the grain size can indicate the influence of FR on the grain size. This further supports the method of selecting grain characteristics which are able to reveal the penetration performance.

In summary, the research in this paper is carried out through the following steps. In Step I, the minimum radial reduction value of the hammer die is calculated based on the IRT method at a given FR of 64%. Based on the identified minimum radial reduction value obtained in Step I (i.e., 9.02 mm), in Step II, a safety value higher than the minimum radial reduction value is determined to ensure a thorough penetration performance, reaching the FR value of 75%. In Step III, simulations are carried out for the RF process with different temperatures and FRs designated in Section 2.2, where the optimal process parameters for the RF process are obtained. In Step IV, the experiment of the RF process is performed based on the identified optimal operating parameters. In Step V, samples are cut from the central and edge positions of both the RF-deformed blank and the raw material. In Step VI, samples from Step V are treated by the SSIHT process. In Step VII, microstructure analysis is carried out for the treated samples from Step VI, where the AGS value of samples can be obtained. The difference in the AGS values is used to quantitatively indicate the penetration performance of the RF-deformed blank.

## 3. Results

### 3.1. Simulation Results and Discussion for the Radial Forging Process

#### 3.1.1. Effect of FR on the RF Process

**Figure 6:**
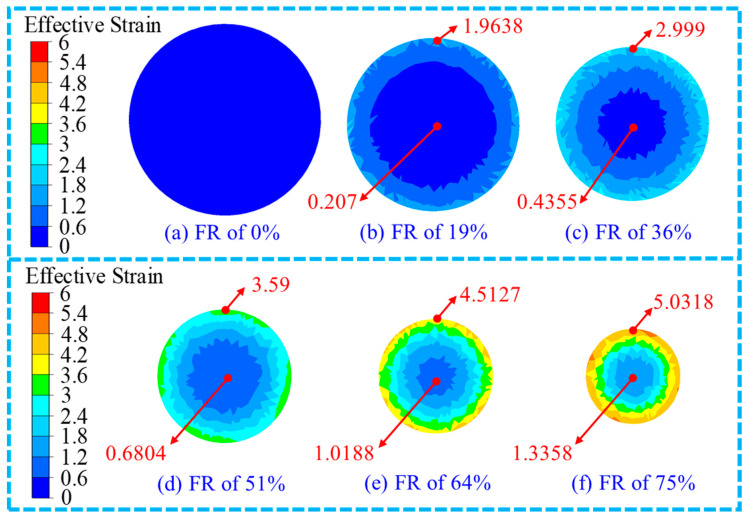
Effective strain distribution of the RF-deformed blank with different RFs at room temperature.

The penetration of the material means the depth of the deformation along the radial direction, as the design of the radial forging machine needs to satisfy the length requirement of the product. Therefore, the strain distribution in the cross-section can be regarded as independent from length. Therefore, in this study, the strain distribution in the cross-section is provided and analysed. The distribution of the effective strain in the cross-section at the middle position along the length direction of the RF-deformed blank with different FRs at room temperature is shown in Figure 6. The maximum effective strain in the cross-section of the RF-deformed blank was found at the edge of the cross-section at different FRs while the minimum value was at the centre of the cross-section. In other words, the effective strain decreased from the edge to the centre position of the RF-deformed blank. The effective strain at the edge position of the RF-deformed blank was significantly larger than that at the centre position. This is mainly because the material deformation degree at the edge position was larger compared to the centre region during the RF process. The effective strain at the edge position was found to be 1.9638, 2.999, 3.59, 4.5127 and 5.0318 when the FR was 19%, 36%, 51%, 64% and 75%, respectively, with that at the central position being 0.207, 0.4355, 0.6804, 1.0188 and 1.3358. Therefore, the effective strain at both the edge and centre positions increased with an increase in the FR, which can be seen in Figure 6. According to the investigation from Du et al. [18], the increase in FR improved the forging penetration efficiency, which helped to reduce the difference in the effective strain between the centre position and the edge position of the RF-deformed blank. Moreover, the results shown in Figure 6 are also consistent with the results reported by Wang et al. [28]. Additionally, the internal effective strain of the RF-deformed blank was not less than 0.2 under the forging rule reported by Liu et al. [29]. The lowest effective strain in this study was 0.207, indicating that the RF-deformed blank with an FR of 19% can be considered to be completely forged.

**Figure 7:**
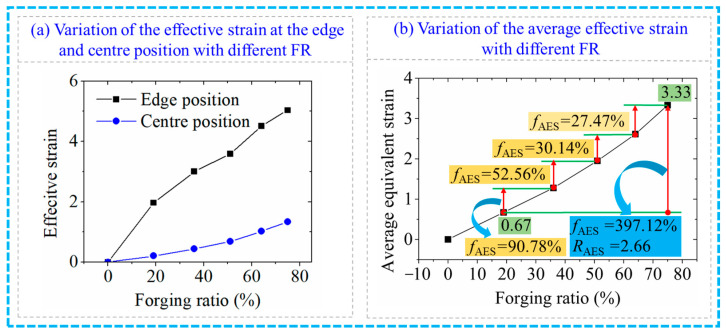
Variation of the effective strain with the FR.

Figure 7 shows the variation in effective strain with different values of FR in the RF process for the 6063 wrought aluminium alloy. As presented in Figure 7a, the effective strain at both edge and centre positions increased with an increase in the FR. However, the increasing trend of the effective strain at the edge position slowed down when the FR was higher than 19%, indicating that the microstructure at the edge position had been fragmented and became relatively compressed. Thus, the influence of increasing the FR on the compactness of the microstructure at the edge of the RF deformation gradually reduced. The effective strain at the centre position increased with the increase in the FR, which indicated that the deformation degree of the centre position of the RF-deformed blank increased when increasing the FR. Therefore, it can be concluded that the increase in FR is helpful to improve the penetration performance of the RF-deformed blank. Figure 7b shows the variation trend of the average effective strain (AES) with different RF. The AES is calculated from the average of all the effective strains shown in Figure 6. As shown in Figure 7b, AES increased with the increase in FR. The AES was found as 0.67 and 3.33 when the FR was 19% and 75%, respectively. The increment of the AES was 2.66 with an improvement ratio (*f*_AES_) of 397.12%. Moreover, with the increase in FR from 19% to 36%, 36% to 51%, 51% to 64% and 64% to 75%, the *f*_AES_ was obtained as 90.78%, 52.56%, 30.14% and 2.47%, respectively. Therefore, the penetration performance of the RF-deformed blank can be improved by increasing FR, yet the *f*_AES_ decreases.

**Figure 8:**
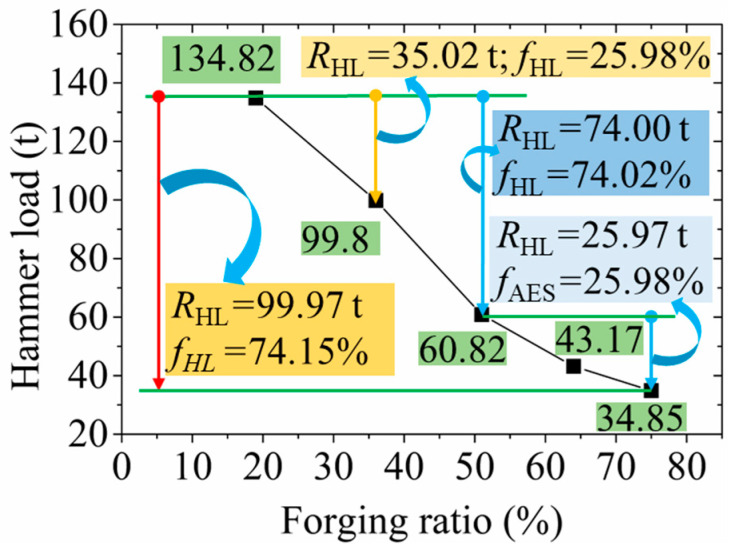
Variation of the hammer load with the FR.

The variation curve of the hammer load with the FR in the RF process for the 6063 wrought aluminium alloy is depicted in Figure 8. The hammer load decreased rapidly at first and then slowly when FR rose, which can be attributed to two factors including the deformation temperature increasing and the contact area decreasing. Firstly, according to the investigation results reported by Zou et al. [17], the temperature increased with an increase in the FR. Namely, under the action of the high-frequency forging of the hammer die in the RF process, the deformation temperature of the RF-deformed blank increased rapidly, resulting in enhanced material fluidity. Consequently, the deformation resistance of the forging was reduced, so the hammer load decreased rapidly. Secondly, with the increase in FR, the diameter of the forging decreased. Therefore, under the same radial reduction, the decrease in the area of blank actually contacted by the hammer die in the RF process was obtained, causing a decline in the hammer load. The hammer load required was found to be 134.82 t, 99.8 t, 60.82 t, 43.17 t and 34.85 t when the FR was 19%, 36%, 51%, 64% and 75%, respectively. The decrease in hammer load was found to be 35.02 t when the FR increased from 19% to 36%, where the reduction ratio of the hammer load (*f*_HL_) was 25.98%. The reduction in the hammer load was 99.97 t with the FR increasing from 19% to 75%, along with a reduction ratio of *f*_HL_ at 74.15%. Moreover, with the increase in FR from 19% to 51% and 51% to 75%, the reduction in hammer load was observed as 74.00 t and 25.97 t, respectively, with the *f*_HL_ of 74.02% and 25.98%, respectively. Therefore, when the FR exceeded 51%, the reduced trend of the hammer load slowed down. This was because the densification degree of the RF-deformed blank was quite high at that level of FR, resulting in the reduction degree of the hammer load caused by the deformation temperature increasing and deformation volume decreasing.

#### 3.1.2. Effect of Blank Temperature on the RF Process

**Figure 9:**
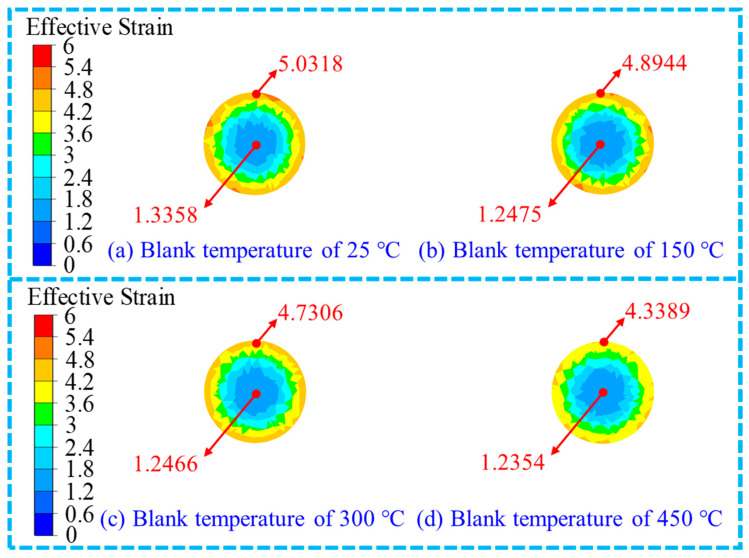
Effective strain distribution of the RF-deformed blank with the FR of 75% and different blank temperatures.

The results of the distribution of the effective strain in the cross-section and middle position of the RF-deformed blank of the metal blank in the RF process with the FR of 75% at different blank temperatures are shown in Figure 9. The distribution pattern under different blank temperatures was found to be significantly similar. Under different blank temperatures, the effective strain at the edge position was all larger than that at the centre position, which was consistent with the results reported by Liu et al. [29] and Li et al. [26]. As shown in Figure 9a, when the blank temperature was 25 °C, the relatively large effective strain marked with red and orange was distributed locally at the edge position, while the small values of effective strain marked with light blue were distributed at the centre position. When the deformation temperature rose to 150 °C and 300 °C as shown in Figure 9b,c, the large effective strain marked with red disappeared, while the area of effective strain marked with earthy yellow at the edge position decreased. Moreover, there was no obvious difference between the areas of effective strain marked with light blue at the centre position. When the blank temperature was 450 °C, the area of the effective strain marked with earthy yellow obviously decreased while that of the effective strain marked with yellow increased. The effective strain area marked in light blue at the centre remained basically unchanged, as shown in Figure 9d.

**Figure 10:**
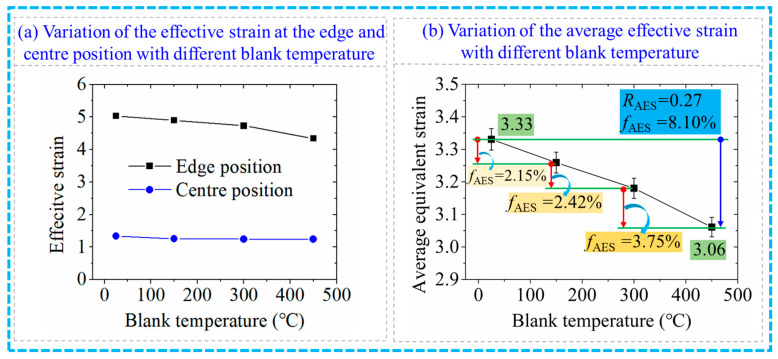
Variation of the effective strain with the blank temperature.

The change in the effective strain under different blank temperatures in the RF process for the 6063 wrought aluminium alloy is presented in Figure 10. Compared with the variation curve of the effective strain with the FR illustrated in Figure 7a, the effect of blank temperature on the effective strain was not obvious. With the increase in the blank temperature, the effective strain at the edge position decreased slightly, while the change degree of the effective strain at the centre position was not obvious. The slight decrease in the effective strain at the edge position may be attributed to the increased flow deformation capacity of the material with the increase in blank temperature. As illustrated in Figure 10b, the AES declined when the blank temperature elevated. The AES was obtained as 3.33 and 3.06 when the blank temperature was 25 °C and 450 °C, respectively. The reduction in the AES (*R*_AES_) was 0.27 with a reduction ratio (*f*_AES_) of 8.10%. Moreover, the *f*_AES_ was found to be 2.15%, 2.42% and 3.75% with the increase in the blank temperature from 25 °C to 150 °C, 150 °C to 300 °C and 300 °C to 450 °C, respectively. 

**Figure 11:**
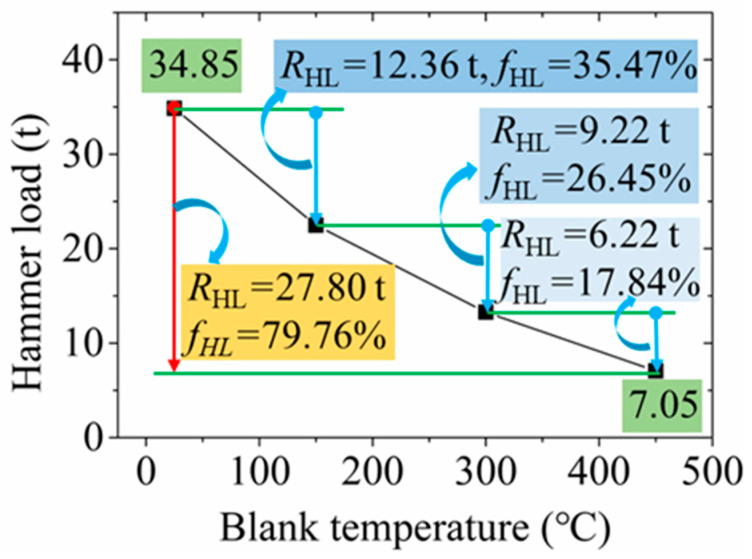
Variation of the hammer load with the blank temperature.

The variation curve of the hammer load with different blank temperatures in the RF process for the 6063 wrought aluminium alloy is shown in Figure 11. With an increase in blank temperature, the hammer load decreased. The hammer load was obtained as 134.82 t, 60.82 t, 43.17 t and 34.85 t when the blank temperature was 25 °C, 150 °C, 300 °C and 450 °C, respectively. The reduction in hammer load was observed as 27.80 t when the blank temperature increased from 25 °C to 450 °C with a reduction ratio of hammer load (*f*_HL_) of 79.76%. The reduction in hammer load was found to be 12.36 t, 9.22 t and 6.22 t and the *f*_HL_ was 35.47%, 26.45% and 17.84% with the increase in blank temperature from 25 °C to 150 °C, 150 °C to 300 °C and 300 °C to 450 °C, respectively, indicating that the reduced trend of hammer load slowed down with the increase in blank temperature. Therefore, the blank temperature was an important factor in the reduction in the hammer load. A small forging force was required when the blank temperature was high, which can provide a reference for reducing the requirements on the forging tonnage of equipment.

According to the analysis of the RF plastic deformation process shown in Figure 6, Figure 7, Figure 8, Figure 9, Figure 10 and Figure 11, it can be concluded that with an increase in FR, the effective strain at the edge and the centre positions of the RF-deformed blank gradually increased, but the increasing trend of the effective strain at the edge gradually slowed down, where the hammer load decreased rapidly first and then slowly. With an increase in blank temperature, the effective strain at the edge position decreased slightly, while the change in the degree of the effective strain at the centre position was not obvious. However, the hammer load is significantly reduced with increasing the blank temperature; that is, the requirements for forging tonnage of the RF machine would be reduced. A qualified penetration performance of the RF process needs to have a relatively large effective strain and relatively uniform effective strain distribution. Therefore, within the allowable range of tonnage capacity of the RF machine, the selection principle of process parameters for the RF process of the wrought aluminium alloy can be summarised as follows: process parameters with low temperature and large FR should be selected as far as possible. 

The SXP-16 RF machine with a maximum forging force of 200 t is used in this work, which can completely meet the required forging force of 34.85 t for the RF process of the 6063 wrought aluminium alloy blanks at room temperature. In addition, the heating process of the metal blank is not required when the RF process is operated at room temperature, which can decrease the forging cost and improve forging efficiency. Furthermore, it has been found that the reduced trend of hammer load slowed down when the FR exceeded 51%. With the increase in FR from 51% to 64%, the reduction in hammer load was 17.65 t. The reduction in the hammer load was 8.32 t when the FR increased from 64% to 75%, which was relatively low. However, when the FR increased by 75%, extra energy consumption was needed for a further FR increase. Thus, the FR of 64% was regarded as an appropriate process parameter for the RF process due to the consideration of reducing energy consumption. In summary, the appropriate process parameters for the RF process in this work were selected as an FR of 64% and a blank temperature of 25℃.

### 3.2. Experimental Results and Discussion for the Radial Forging Process

**Figure 12:**
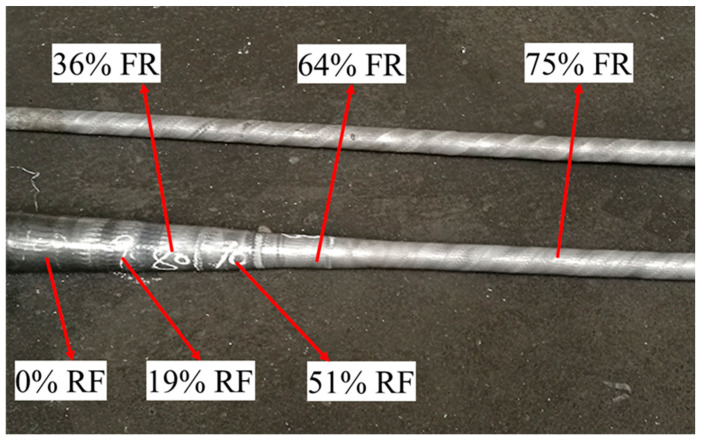
Micro-morphology of the RF-deformed blank with different FR at room temperature.

Figure 12 shows the macro-morphology of the RF-deformed blank with different FRs at room temperature. The diameter of the RF-deformed blank gradually decreased with the increase in the FR. The RF-deformed blank with different FRs had high-quality forming shapes and smooth blank surfaces, indicating the RF-deformed blanks can be successfully produced via the RF process with the appropriate process parameters obtained by the simulation.

**Figure 13:**
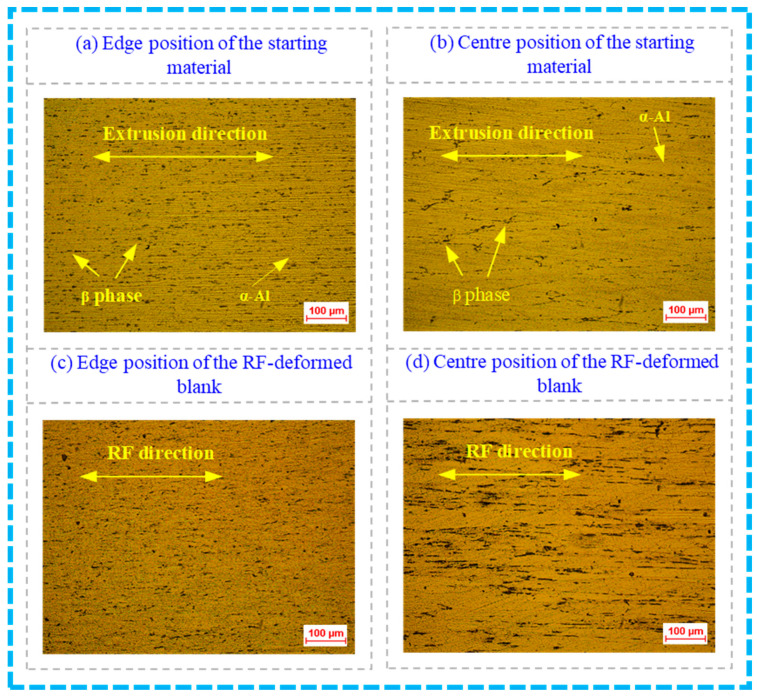
Longitudinal section microstructures of the starting material and the RF-deformed blank with an FR of 64% at room temperature.

The longitudinal section microstructures from different positions of the starting material and the RF-deformed blank with an FR of 64% at room temperature are presented in Figure 13, which can show the penetration in a qualitative way. As illustrated in Figure 13a,b, the microstructure of the starting material mainly included the solid solution of Mg in Al (α-Al) and the β phase (Mg_2_Si), where the β phase was located discontinuously and mainly distributed along the grain boundary. Moreover, the microstructure at the edge and centre positions of the starting material had a certain directivity in the direction of the extrusion. This was because of the application of a commercial 6063 wrought aluminium extruded blank as the starting material in this work. However, the deformation microstructure at the edge position was more compressed than that at the centre position, which was mainly because the starting wrought aluminium bar was produced by the extrusion process.

Changes in the microstructures of the RF-deformed blank shown in Figure 13c,d can be observed compared to those in Figure 13a,b. The deformation microstructure at the edge position of the RF-deformed blank became compressed, forming a large number of closely arranged fibrous microstructures. The grains at the centre position were visibly fractured. The entire microstructure was significantly elongated along the direction in the radial forging due to the plastic deformation in the RF process.

**Figure 14:**
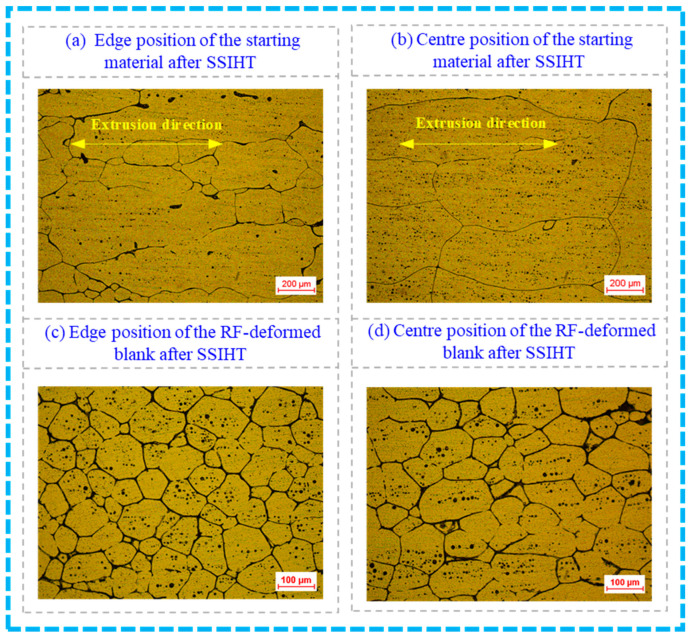
Microstructures of the starting material and the RF-deformed blank after IHT at 630 °C for 10 min.

Figure 14 shows the longitudinal section microstructure from different positions of the starting material and the RF-deformed blank after the IHT process at 630 °C for 10 min. As shown in Figure 14a,b, when the starting material had been conducted by the SSIHT process at 630 °C for 10 min, a certain directionality along the extrusion direction of the starting aluminium alloy blank was observed in the longitudinal section microstructure at the edge and centre positions of the semi-solid material. Meanwhile, the solid grain was relatively large and the spherical degree of the solid grain was low. However, the size of the solid grain at the edge position was obviously smaller than that in the central region. This was mainly because the diameter of the 6063 aluminium alloy blank in the initial extrusion state was relatively large and the extrusion deformation was small and uneven. Therefore, the strain energy obtained by the extrusion forming process of the starting 6063 aluminium alloy blank was low, resulting in poor grain refinement and spherical degree of semi-solid material prepared by the SSIHT of the starting material. As shown in Figure 14c,d, the grain size at both the edge and the centre positions significantly reduced when the FR is 64%, where the spherical degree of solid grain obviously improved with an excellent homogeneity in the solid grain. The above phenomena can be explained as follows.

A large amount of distortion energy was stored in the RF-deformed blank in the form of vacancy, dislocation or lattice defects after the starting aluminium alloy blank was deformed in the RF process [9,21,30,31]. In the subsequent SSIHT process, the distortion energy provided the driving force for recrystallisation. The critical dimension of the recrystallisation nucleation r* is expressed by Equation (7) [32]

(7) $$r^{*}=\frac{2r}{\Delta E_{S}}$$

where $\Delta E_{S}$ is the change in distortion energy per unit volume and r is the interface energy per unit area.

It can be seen from Equation (6) that ΔEs decreases when the deformation energy storage during the RF process increases, which then causes the reduction in the r*. According to the investigated results reported by Moshtaghi et al. [8], the dislocation density increases with an increase in the deformation of the cold-rolled Al-Zn-Mg-Cu alloy. Therefore, the dislocation density of the RF-deformed blank increases with the increase in FR. Namely, the deformation energy stored in the RF-deformed blank with the FR of 64% was more than that in the starting material in this work. Therefore, a small recrystallisation grain size would be obtained under a large deformation, which helps to obtain a finer and spherical semi-solid material in the subsequent SSIHT process.

According to the quantitative analysis of the semi-solid microstructure shown in Figure 13, the improvement in the penetration performance of the RF process for the wrought aluminium alloy can be calculated, which is summarised in Table 3. The AGSs of the microstructure shown in Figure 13a,b were obtained as 294.39 μm and 480.82 μm, respectively. The difference between the AGSs at the edge and centre positions was found as 186.43 μm. However, the AGSs of the microstructure shown in Figure 13c,d were observed as 64.11 μm and 79.19 μm, respectively. The difference between the AGS at the edge and centre positions was 15.09 μm. Therefore, compared with the starting material used in this work, the improvement ratio of the penetration performance for the RF-deformed blank with the FR of 64% and the blank temperature of 25 °C was obtained as 91.19%.

## 4. Conclusions

(1) With the increase in FR, the effective strains at the edge and the centre positions of the RF-deformed blank gradually increase. With an increase in the FR from 19% to 36%, 36% to 51%, 51% to 64% and 64% to 75%, the *f*_AES_ is obtained as 90.78%, 52.56%, 30.14% and 2.47%, respectively. The reduction in hammer load is 99.98 t with an increase in the FR from 19% to 75%, along with a reduction ratio of 74.15%. Moreover, with the increase in the FR from 19% to 51% and 64% to 75%, the reduction in hammer load was observed as 74.00 t and 25.98 t, respectively.
(2) With an increase in blank temperature, the effective strain at the edge position decreases slightly, while the change degree of the effective strain at the centre position is not obvious. The *f*_AES_ is 2.15%, 2.42%, and 3.75% with the increase in the blank temperature from 25 °C to 150 °C, 150 °C to 300 °C and 300 °C to 450 °C, respectively. The hammer load significantly reduces when increasing the blank temperature. The reduction in hammer load is found to be 12.36 t, 9.22 t and 6.22 t with changes in blank temperature from 25 °C to 150 °C to 300 °C and 300 °C to 450 °C, respectively.
(3) The selection principle of process parameters for the RF process of the wrought aluminium alloy is concluded as follows: process parameters with low temperature and large FR should be selected as far as possible. The appropriate process parameters for the RF process in this work are then selected as an FR of 64% and a blank temperature of 25 °C.
(4) The semi-solid microstructure with the AGS of 71.65 μm can be obtained by applying the SSIHT process to an RF-deformed blank at 630 °C for 10 min. Compared with the starting material, the improvement ratio of the penetration performance is obtained as 91.19% for the RF-deformed blank with an FR of 64% and a blank temperature of 25 °C.

## Figures and Tables

**Table 1:** Specifications of chemical elements (wt.%).

Si	Fe	Cu	Mn	Mg	Zn	Ti	Cr	Al
0.38	0.15	0.023	0.018	0.65	0.02	<0.1	<0.1	Bal.

**Table 2:** Process parameters in simulations for RF.

Constant Process Parameters	Specific Values	
Dimension of the starting material	A commercial 6063 wrought aluminium extruded bar with 100 mm in diameter and 1000 mm in length under T6 state	
Radial reduction value	10 mm/stroke	
Frequency for RF	580 stroke/min	
Feed speed of hammer die	50 mm/s	
Feed speed of blank	580 mm/min	
Rotation speed of blank	19.3 r/min	
Relative feed rate	1 mm/stroke	
Relative angle of rotation	12 °/stroke	
Variable process parameters		
Group No.	FR (RF-deformed diameter)	Blank temperature
1	19% (90 mm), 36% (80 mm), 51% (70 mm), 64% (60 mm), 75% (50 mm)	25 °C
2	75% (50 mm)	25 °C, 150 °C, 300 °C, 450 °C

**Table 3:** Improvement of penetration performance of the RF process.

Process Method	AGS at the Edge Position (μm)	AGS at the Centre Position (μm)	Difference	Improvement Ratio
IHT of the starting material	294.39	480.82	186.43	91.19%
IHT of the RF-deformed blank	64.10	79.19	15.09	

## Data Availability

The data presented in this study are available on request from the corresponding author. The data are not publicly available due to project confidentiality requirements.
